# Completely Isolated Retroperitoneal Enteric Duplication Cyst with Adenocarcinoma Transformation Managed with Robotic Radical Nephrectomy

**DOI:** 10.1089/cren.2017.0016

**Published:** 2017-03-01

**Authors:** Kassem Faraj, Luke Edwards, Alia Gupta, Brian Seifman

**Affiliations:** ^1^ Department of Urology, Oakland University William Beaumont School of Medicine, Rochester, Michigan.; ^2^ Department of Urology, Beaumont Health System, Royal Oak, Michigan.; ^3^ Department of Pathology, Beaumont Health System, Royal Oak, Michigan.; ^4^ Michigan Institute of Urology, Troy, Michigan.

**Keywords:** adenocarcinoma, mucinous, kidney, nephrectomy, robotic

## Abstract

***Background:*** Enteric duplication cysts are congenital malformations that typically affect children in infancy, but can also affect adults. Rarely, these cysts can be complicated by malignancy. We present the first case of retroperitoneal duplication cyst that was complicated by malignancy transformation and managed by robot-assisted excision.

***Case presentation:*** A 64-year-old female with a history of a left-sided renal cyst presented with a 4-month history of abdominal pain and fatigue. MRI revealed a bilobed cyst, with components measuring 6.9 × 6.6 and 6.1 × 6.9 cm, which had grown since previous imaging, and hemorrhage in some portions of the cysts, as well as cystic wall enhancement, suggesting a possible malignancy. The patient consented to a robot-assisted partial (possible radical) nephrectomy. During the procedure, the cystic structure appeared to have grown since imaging, was intimately associated with the hilum, and had a complex vasculature, which prompted us to perform a radical nephrectomy. Grossly, the specimen consisted of a 14.8 cm cystic structure at the superior portion of the kidney, but was not contained within the renal parenchyma. Histologically, the internal mucosa of the cyst showed columnar epithelium with high-grade dysplasia and carcinoma *in situ* with focal individual cell infiltration into the superficial portion of the inferior part of the cyst. The patient saw a medical oncologist and was instructed to follow up with quarterly imaging to assess for disease progression.

***Conclusion:*** Enteric duplication cysts are uncommon entities that can occur in various locations in the body, causing a wide spectrum of symptoms, and are rarely complicated by malignancy transformation. Robot-assisted surgical resection is an option that we have shown to be effective in managing these patients.

## Introduction and Background

Enteric duplication cysts are congenital malformations that are typically attached or adjacent to a wall of the gastrointestinal tract and can share a common blood supply with the native bowel.^1^ They most commonly affect the ileocecal region, but have been reported to involve the stomach, mesentery, and colon. Presentation can include asymptomatic, abdominal pain, gastrointestinal bleeding, and intestinal obstruction.^1^ Duplication cysts typically present in children; however, cases affecting adults have also been reported.^2^ These cysts can also be completely separate from the bowel and have their own blood supply and rarely can be found near retroperitoneal structures.^3^ A rare complication of these cysts is malignancy transformation, whereby adenocarcinoma has been reported.^4^ Herein, we present the first case to our knowledge of a retroperitoneal enteric duplication cyst complicated by mucinous carcinoma that was managed through robot-assisted radical nephrectomy.

## Presentation of Case

**FIG. 1.**
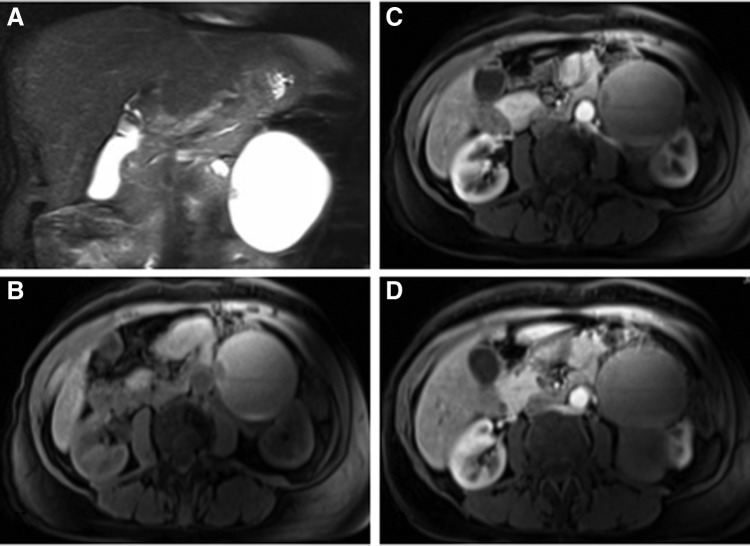
Abdominal MRI. T2 phase coronal section **(A)** illustrating a cystic structure measuring 6.9 × 6.6 cm in the left retroperitoneum with an area of nodularity medially. Axial sections in the T1 phase before **(B)** and after **(C)** gadolinium contrast through the medial nodular structure showing subtle nodular and cystic wall enhancement. Axial section in the T1 phase after contrast illustrating that two cystic structures are associated with the left kidney **(D)**.

A 64-year-old female with a history of kidney stones and renal cysts presented with a 4-month history of abdominal pain and fatigue. Her pain was vague and localized to the left lower quadrant. A CT scan without contrast was initially performed, which showed a bilobed cystic structure, with components measuring 6.9 × 6.6 and 6.1 × 6.9 cm associated with the left kidney. These structures had shown interval growth since the last study, 5 years prior. Subsequent MRI (Fig. 1) revealed hemorrhage in some portions of the cysts, as well as cystic wall and nodular enhancement, suggesting a possible malignancy. The patient was counseled on the risks of malignancy, based on the MRI findings, and a robotic partial (possible radical) nephrectomy was recommended.

After obtaining the patient's informed consent, general anesthesia was induced and the patient was placed in the right lateral decubitus position. Four total robotic ports were placed: 8-mm camera port lateral to the umbilicus, 8-mm left-handed arm several fingerbreadths subcostally just lateral to the midline, 8-mm right-handed arm mid way between the camera port and the left anterior superior iliac spine, and 12-mm assistant port in the midline infraumbilical location. Once the robot was docked, the line of Toldt was incised and the colonic mesentery was reflected off the kidney. The large mass was immediately observed and appeared to have grown since imaging was done 5 months prior. A plane was then made between the mass and colon. The hilum was identified, as was the gonadal, which inserted into the renal vein. One portion of the mass appeared to abut the hilum. A plane was then developed between the lower pole of the kidney and the psoas fascia. We then skeletonized the renal vein, as well as two renal arteries (one of which inserted into the cystic mass). Gerota's fascia was incised and the kidney was then defatted. A fair amount of aberrant vasculature between the kidney and mass was apparent. The mass was then separated from the tail of the pancreas and splenic vasculature, although several parasitic vessels were encountered, which were cauterized. Owing to a combination of the mass size, broad association with the renal hilum, and aberrant vasculature, a radical nephrectomy was decided upon. A 45-mm vascular staple load was first used to ligate the renal artery associated with the mass. The remaining renal artery and vein were then ligated using a 65-mm load. The mass was freed from the surrounding structures and the specimen was removed by using a large EndoCatch bag. The adrenal was not identified during the procedure. Estimated blood loss was 50 mL. The patient did well postoperatively and was discharged on postoperative day 3.

**FIG. 2.**
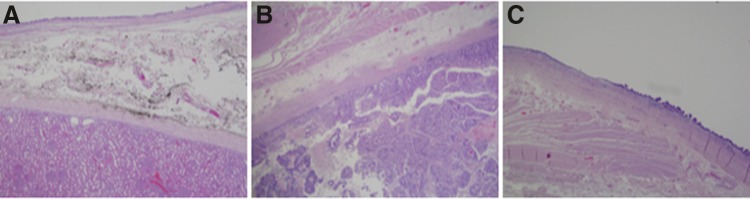
Histopathology from surgical specimen. **(A)** Illustrates normal renal parenchyma (*bottom*) with adjacent cyst wall (*top*) separated by fibroconnective tissue (*middle*). The top of the cyst wall consists of soft intestinal epithelium with an underlying muscular layer. Malignant transformation of the cyst wall into adenocarcinoma **(B)** is shown with neoplastic glands depicted on the *bottom* of the image. Transformation of normal intestinal to dysplastic epithelium can be seen in **(C)**.

Grossly, the specimen consisted of a 14.8 cm cystic structure at the superior portion of the kidney, but was not contained within the renal parenchyma. The cyst was separated by a septum into the superior portion up to 8.2 cm. The lower portion had a size up to 8 cm. Histologically (Fig. 2), the internal mucosa of the cyst showed columnar epithelium with high-grade dysplasia and carcinoma *in situ* with focal individual cell infiltration into the superficial portion of the inferior part of the cyst. These neoplastic components stained positively for PAX8, CDX2, CK7, CK20, P504s, p53, and KIM1. These findings were consistent with a diagnosis of a completely isolated enteric duplication cyst with mucinous adenocarcinoma.

The patient followed up with a medical oncologist, who advised quarterly abdominal CT scans for the next 2 years. Pathologic specimens and subsequent imaging (CT scan of the chest) suggested no regional or distant spread of disease.

## Discussion and Literature Review

Enteric duplication cysts are uncommon congenital entities that most often occur in children. These cysts often present in infancy, as larger studies have consistently reported that affected patients are typically around 2 years of age or younger.^1^ These cysts typically cause symptoms, some of the more common being abdominal pain, vomiting, and constipation. However, because these cysts can occur in various locations in the body, they may also cause respiratory distress or hematuria, when associated with the thorax or the genitourinary system, respectively.^3^ Although most cysts present early in childhood, cases occurring in adults have also been reported. Interestingly, in the rare scenario that there is malignancy transformation of the cyst, these cases are known to occur solely in adults.^4^

Our patient was known to have a “renal cyst” at least 5 years prior, which was interpreted as a benign finding on imaging. It was not until she presented with pain and fatigue that a follow-up MRI was able to demonstrate some growth in the cyst and cyst wall enhancement, suggesting malignancy. This is the first case to describe a duplication cyst in the retroperitoneum that was complicated by malignancy transformation. It is also the first nonesophageal duplication cyst that has been managed through robotic assistance. In symptomatic cases reported in the literature, surgical resection has been effective in alleviating symptoms.^1^ For patients with cysts that have been complicated by malignancy transformation, there is not a standard treatment approach, as some patients have been solely managed by surgical resection, whereas others have, in addition, received chemotherapy.^4^ Although reported outcomes have been generally favorable across these groups, no standard of care has been established for these patients because of its rarity and variable extent of disease. The patient described in this report did not receive chemotherapy and has been instructed to follow up with quarterly imaging to assess for disease progression.

## Conclusion

Enteric duplication cysts are uncommon entities that typically occur in children, but can affect adults. These cysts can occur in various locations in the body, causing a wide spectrum of symptoms, and are rarely complicated by malignancy transformation. Management of these patients typically includes surgical resection and potentially adjuvant chemotherapy. Robot-assisted surgical resection is an option that we have shown to be effective in managing these patients.

## Abbreviations Used

CT: computed tomography
MRI: magnetic resonance imaging

## References

[1] Bower RJ, Sieber WK, Kiesewetter WB Alimentary tract duplications in children. Ann Surg 1978;188:669–674 718292 10.1097/00000658-197811000-00015 PMC1396776

[2] Kim SK, Lim HK, Lee SJ, et al. Completely isolated enteric duplication cyst: A case report. Abdom Imaging 2003;28:12–14 12483377 10.1007/s00261-001-0138-0

[3] Bal HS, Kisku S, Sen S, et al. A retroperitoneal enteric duplication cyst communicating with the right upper ureter in an infant. BMJ Case Rep 2014; pii: 10.1136/bcr-2013-202449 PMC4025355 24813198

[4] Zheng J, Jing H Adenocarcinoma arising from a gastric duplication cyst. Surg Oncol 2012;21:e97–e101 22456198 10.1016/j.suronc.2012.03.002

